# How and why do we screen for colorectal cancer?

**DOI:** 10.25122/jml-2021-0192

**Published:** 2021

**Authors:** Diana Chetroiu, Corina-Silvia Pop, Petruta Violeta Filip, Mircea Beuran

**Affiliations:** 1. Department of Medical Oncology, Bucharest Emergency University Hospital, Bucharest, Romania; 2. Department of Internal Medicine and Gastroenterology, Bucharest Emergency University Hospital, Bucharest, Romania; 3. Department of Surgery, Bucharest Emergency Clinical Hospital, Bucharest, Romania

**Keywords:** colorectal cancer, epidemiology, screening, colonoscopy, quality

## Abstract

After almost 50 years of data analysis, screening for colorectal cancer has proven to be an effective tool in reducing colorectal cancer mortality. However, implementing the optimal strategy represents a challenge for many healthcare facilities around the world. There is much discussion regarding how screening should be done, the optimal tools that should be used, and the proper timing for screening procedures. Another essential step is to maintain the adherence of patients to screening programs. Also, the recommendation for lowering the age to initiate screening is in progress, as there is an increase in colorectal incidence in people born after 1970.

## Introduction 

What is screening, and why do we need screening after all? The term screening as we know and use it today was first used in 1968 when Wilson and Jungner wrote a statement for the World Health Organisation explaining what should be understood by this term and method [1]. At the same time, they published ten principles of screening that are still guiding screening practice (Table 1). All these principles remained the core for future definitions and recommendations.

**Table 1.** Wilson and Jungner’s principles of screening [1].

**The condition should be an important health problem.**
**There should be an accepted treatment for patients with recognized disease.**
**Facilities for diagnosis and treatment should be available.**
**There should be a recognizable latent or early symptomatic phase.**
**There should be a suitable test or examination.**
**The test should be acceptable to the population.**
**The natural history of the condition, including development from latent to declared disease, should be adequately understood.**
**There should be an agreed policy on whom to treat as patients.**
**The cost of case-finding (including a diagnosis and treatment of patients diagnosed) should be economically balanced in relation to possible expenditure on medical care as a whole.**
**Case-finding should be a continuous process and not a “once and for all” project.**

Screening is the process of discovering individuals who are at risk for a certain condition from the general population and separating them from people that do not have that specific condition in order to apply a certain treatment or intervention in order to reduce mortality or morbidity from that specific disease within the population [2].

Screening is not to be mistaken for early detection, as screening applies to a large number of people, with no symptoms, in order to detect a common condition. Early detection is targeted for people with specific symptoms of a particular disease [3].

It has been demonstrated that diet and lifestyle play an important role in colorectal cancer development. However, the exact cascade is still under continuous investigation, as the foremost targeting factors are still unknown. Nevertheless, the role of external factors is definitely demonstrated by the difference between colorectal cancer incidence in developed countries and developing ones that can range up to 10-fold. [4]. Studies of immigrants revealed that after moving from a low-incidence to a high incidence nation, one would have the same risk of developing colorectal cancer as in the adopted nation after one generation [5]. Considering these facts, are we positive that only diet can influence colorectal cancer pathology that much between different countries? Are diet and sedentarism the incriminating factors for colorectal cancer increasing incidence in people younger than 50 years of age?

Until we have the answers to these questions, it is wise to focus on screening programs and colorectal cancer prevention. The five-year survival rate for stage I colon cancer is 91%, but it drops to 72% for locally advanced disease and 14% for stage IV [6]. By analyzing this data, the importance of colon cancer prevention programs is undoubted.

Studies conducted so far demonstrated that the benefit of colorectal screening in preventing specific deaths is between 25% and 50% [7].

## Material and Methods

**Figure 1.**
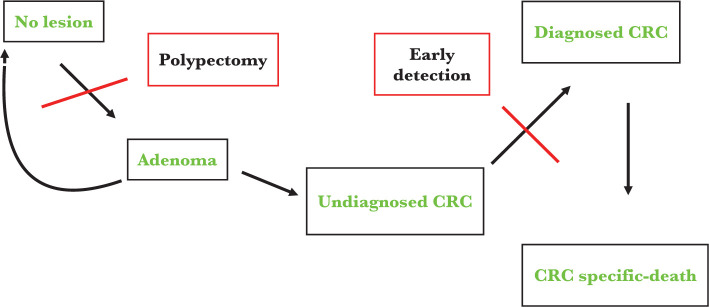
Natural history of colorectal cancer and the role of screening in colorectal cancer (CRC)-specific death [12].

There is a certain variation in colorectal cancer screening guidelines, and we searched the literature to shed light on what we know so far. Colorectal cancer is diagnosed between 65 and 74 years, with a median age at the time of death from colorectal cancer of 73 years [8]. The American College of Physicians (ACP) stated that colorectal cancer screening should start for healthy adults at 50 years of age, until 75 years [9]. Also, the US Preventive Services Task Force (USPSTF) recommended screening for colorectal cancer in adults aged 50–75 (recommendation A) [10]. In 2021, USPSTF updated the recommendations and included adults aged 45–49 (recommendation B) [11]. In order to support this update of colorectal screening recommendations, a model-based program to evaluate de benefits, the impairment, and years gained by individuals from screening programs was created. Starting from the natural history of colon cancer from adenoma to a malignant tumor, two important points were identified in order to prevent colon cancer development or colon-cancer-specific death (Figure 1).

Interestingly, not all adenomas will develop into colon cancer. It usually takes up to 10 years to evolve from an adenoma to a malignant tumor [13]. Therefore, we can say that we have a good window of opportunity to diagnose precursor lesions of colon cancer. Nevertheless, by analyzing this diagram, it is obvious and relatively simple to observe the critical role of screening in preventing colorectal cancer (CRC)-specific deaths. Using this simulation model, it was demonstrated that colorectal cancer screening is efficient for people aged 45 to 75 years old. Little benefit was demonstrated after this interval [12]. Also, The American Cancer Society made a clear statement that colorectal cancer screening should start at 45 years old [14]. In Europe, the European Commission published in 2017 a document stating the importance of screening and validated the decision of most countries to initiate screening in apparently healthy adults from 50 years of age until 74 years [15]. Most of these societies suggest that people aged 76–85 years could be screened in an individualized manner, taking into account the life expectancy and the capability to tolerate treatment if needed. Also, screening can be stopped after 85 years [10, 11, 14, 15].

## Epidemiology of Colorectal Cancer

In the United States of America, colorectal cancer is still the second cause of cancer deaths [16]. Because of screening programs, between the years 2000 and 2010, a decline of 3.3% annually was observed in the incidence of colorectal cancer for people ages 65 years and older [17]. Moreover, this decline continued in the following years [18]. An important piece of data to be mentioned is the increase in the incidence of colorectal cancer that was observed in people aged 50–64 years, with an annually increasing incidence of 1% and by 2% for people younger than 50 years of age [19].

In Europe, data show that approximately 450,000 cases of colorectal cancer are diagnosed every year, and almost half of the patients will die because of this disease [20]. The estimations are that around 1.2 million European citizens live with this diagnosis, according to Globocan [21].

Globally, colorectal cancer accounts for 9.4% of all cancer cases in women, being the second most common cancer diagnosed in women and the third in males, accounting for 10.6% of all cancers. Taken together, approximately 2 million people are diagnosed with colorectal cancer every year [22].

## Screening Tests and Evidence

As it was mentioned before, given the purpose of screening programs to reduce colorectal cancer mortality, it is of utmost importance that these programs comply with international standards, are well organized, and meet all the quality criteria. Each step of a qualitative, well-organized program must be closely monitored and appraised. A clear and well-established outcome for all people involved must be one of the leading purposes.

In order to identify people at risk for colorectal cancer, a population-based call or re-call program is advisable as it is considered a high-quality and standardized approach [23]. As for screening methods, generally speaking, screening tests must fulfill a few essential characteristics: high specificity and sensitivity, applicable for a large population, and not so high costs. Specificity and sensitivity are the characteristics of very accurate tests [2]. Accurate tests are important in order to avoid false-positive or false-negative results. Also, the tests must be easily accepted by the general population and be repeated whenever necessary as they are meant for healthy individuals [24]. No screening test for colorectal cancer is 100% precise, and no method has proved so far to reduce all-cause mortality in screening groups [25].

The latest recommendations are that each screening technique is offered, taking into account a risk-stratified model. In this model, colonoscopy should be the first option if a person has a high risk of developing colorectal cancer (family history of colorectal cancer, diet, obesity, diabetes). On the contrary, if a person has an average risk for CRC, then a stool-based test should be the first option [26].

### Stool blood tests

Fecal occult blood tests (FOBT) efficacy was determined in observational studies, using colonoscopy as the reference method for detecting polyps and colorectal cancer. The results of these studies regarding the sensibility and specificity of FOBT and fecal immunochemical test (FIT) are shown in Table 2.

**Table 2.** Sensibility and specificity of FOBT and FIT [27, 28]. FOBT – fecal occult blood test; FIT – fecal immunohistochemistry test; CRC – colorectal cancer).

**Test**	**Sensibility in detecting polyps**	**Sensibility in detecting CRC**	**Specificity in detecting CRC**
**FOBT**	11- 25%	33- 75%	98-99%
**FIT**	27- 29%	71- 75%	94-95%

Because of the much lower false-positive results rate, FIT is preferred over FOBT [29]. Multitargeted DNA stool analysis (FIT-DNA) implies a molecular analysis of DNA fragments from cancer cells and occult hemorrhage in the stool. Tests conducted so far that compared this method to FIT or FOBT demonstrated that FIT-DNA has a sensitivity greater than 90% in detecting CRC but a lower specificity [30]. Actual recommendations are that FOBT or FIT should be performed each year, and FIT-DNA should be repeated every 1–3 years [31]. If the test is positive, then a colonoscopy should be performed.

The harm of these stool blood tests comes from the need to complete the screening with a colonoscopy in case of a positive result. However, it may be a false-positive result as it is known that FIT-DNA has a lower specificity compared to FIT and, thus, a higher rate of false-positive results [32].

### Flexible sigmoidoscopy and colonoscopy

Flexible sigmoidoscopy was studied as a single method for screening and demonstrated that it could reduce colorectal cancer-specific mortality [32]. When combining flexible sigmoidoscopy with a FIT, the decrease of colorectal cancer-specific mortality rate is even greater [33, 34]. In the presence of a tumor or polyps, it is recommended to complete the investigation with a colonoscopy [35]. Taking into consideration that the prevalence of right-sided colon cancer is increasing, especially in women, it is not considered the best option for screening [36]. The harm of the method comes from the need to perform a new endoscopic method and the risk of perforation or bleeding.

Colonoscopy remains the most important test for colorectal screening. It enables examination of the entire colon, a biopsy of suspected lesions, and polyp resection. The limits of colonoscopy are given by the need for bowel preparation that may lead to dehydration and electrolyte imbalance, the experience of the doctor performing the examination, the presence of an anesthesiologist, and the possible complications of the procedure itself, like perforation and bleeding. It is to be mentioned the difficulty in detecting serrated polyps or flat lesions [37]. Despite all these, colonoscopy has a sensitivity between 88% and 100% in depicting adenomas of at least 1 cm in diameter and more than 95% in detecting malignant tumors [38]. It is the only test that can allow immediate action in preventing colon cancer as it allows the endoscopist to remove the polyp in the same session.

The harms of endoscopy come from gastrointestinal or cardiovascular side effects that can be diagnosed before or after the procedure.

According to the USPSTF and other studies and collaborative models, the optimal interval for screening is determined by the chosen method. The reduction in the mortality rate for each stool-based test and direct visualization test is shown in Table 3 [2, 3, 9, 32, 36, 39].

**Table 3.** Optimal interval for screening methods [2, 3, 9, 32, 36, 39].

**Screening method**	**Interval**	**Reduction in mortality rate**
**FOBT**	Every year	32%
**FIT**	Every year	Unknown
**FIT-DNA**	Every 3 years	Unknown
**Colonoscopy**	10 years	68%
**Sigmoidoscopy**	5 years	27%
**Sigmoidoscopy plus FIT**	Every 10 years plus FIT every year	38%

## Cost-Effectives of Screening

All screening methods presented are considered to be cost-effective compared with no screening [40]. It appears that FIT every year is more cost-effective than sigmoidoscopy every 5 years [41]. Colonoscopy every 10 years is more cost-effective than annual FIT or compared to sigmoidoscopy every 5 years plus FIT every year [41]. By contrast, another study revealed that sigmoidoscopy every 5 years plus annual FIT is more effective and has fewer costs compared to colonoscopy every 10 years [42].

## Cascades – Tooling Up for Screening in Romania

In Romania, the Ministry of Health started in July 2020 the ROCCAS II program for the prevention, early diagnosis and treatment of precursor lesions in colorectal cancer. This project will carry on for 4 years and is addressed to vulnerable people from different regions of Romania in the attempt to fight against poverty, discrimination, and improving access to medical care. The screening will target people between 50–75 years of age, and the method chosen for screening is colonoscopy.

## Conclusion

Efforts should be made in order to increase and maintain a high rate of adherence to screening programs. It is believed that through educational projects, acknowledging the available screening methods will increase compliance in different populations. Choosing an individualized method for different populations might be the answer for increasing acceptance. High-quality standards for screening programs should be strictly followed in order to achieve the best outcomes and established goals.

## Acknowledgments

### Conflict of interest

The authors declare that there is no conflict of interest.
